# Advances in the Detection and Management of Vulnerable Coronary Plaques

**DOI:** 10.1161/CIRCINTERVENTIONS.125.015529

**Published:** 2025-07-28

**Authors:** Marco Spagnolo, Daniele Giacoppo, Claudio Laudani, Antonio Greco, Simone Finocchiaro, Maria Sara Mauro, Antonino Imbesi, Davide Capodanno

**Affiliations:** 1 Division of Cardiology, Azienda Ospedaliero-Universitaria Policlinico “G. Rodolico–San Marco”, University of Catania, Italy.

**Keywords:** cardiovascular diagnostic techniques, cardiovascular drugs, cardiovascular risk factors, coronary artery disease, myocardial infarction, percutaneous coronary intervention, risk factors

## Abstract

Efforts to enhance risk stratification in patients with coronary artery disease have driven the pursuit of early detection of rupture-prone plaques—before destabilization and the onset of life-threatening thrombosis—giving rise to the concept of the vulnerable plaque (VP). Invasive diagnostic modalities closely mirror histology and provide instrumental information on VP hallmarks and their prognostic significance. However, limited positive predictive value and invasive nature restrict their use for systematic screening. Noninvasive techniques offer broader application potential, but their specificity and resolution remain inferior to those of invasive techniques. A deeper understanding of the complex interplay between traditional ischemic risk factors, anatomic settings, rheological effects and systemic influences contributing to plaque evolution and rupture has refined our approach to identifying and managing VPs. Systemic therapies have been shown to counteract plaque progression and stabilize VPs by thickening the fibrous cap, decreasing atheroma and necrotic core volumes, and reducing inflammation. In parallel, the hypothesis of sealing and passivating VPs by intravascular imaging-guided preventive stenting is gaining support after the promising results of clinical trials and substantial advances in contemporary device performance and biocompatibility. Upcoming evidence will be instrumental in defining the net benefit of novel diagnostic tools and therapeutic strategies for VPs.

Ischemic heart disease is a leading cause of mortality and morbidity worldwide.^1^ In the United States, 20.5 million individuals suffer from coronary artery disease, and ≈375 000 deaths are attributable to this condition every year.^2^ In European Society of Cardiology member countries, ≈48 million people suffer from ischemic heart disease, and almost 6 million new cases are diagnosed every year.^3^ Although ischemic heart disease includes several functional and structural conditions involving the epicardial and microvascular components, coronary atherosclerosis remains the predominant type of lesion underlying myocardial ischemia.^4^

The accumulated evidence on atherothrombosis supports the notion that coronary artery disease is a complex and dynamic condition leading to plaque progression and destabilization.^5–8^ Coronary artery disease destabilization, ultimately resulting in plaque rupture or erosion, provides the histopathologic groundwork for most variants of acute coronary syndrome (ACS), primarily spontaneous myocardial infarction.^5–8^ The exposure of tissue factors and collagen fibrils to luminal blood through the disrupted intimal surface triggers platelet-rich thrombosis, provoking flow limitation and myocardial ischemia.^9^ However, the dynamic process of plaque disruption can also be subclinical or mildly symptomatic as a result of the balance between thrombosis-promoting and inhibiting factors.^7,10^ In fact, spontaneous healing of ruptured plaques can lead to layered plaque progression, sometimes eventually evolving into recurrent rupture under precipitating conditions.^11^

The quest to detect rupture-prone plaques before the occurrence of destabilization and life-threatening thrombosis has leveraged the concept of the so-called high-risk or vulnerable plaque (VP).^12,13^ The VP challenges the axiom that greater stenosis correlates with a higher risk of unstable angina and myocardial infarction. Indeed, histopathologic investigations have shown that lipid-core volume has no significant correlation with stenosis degree, and the arterial wall can remodel by expanding its external diameter to accommodate atherosclerotic plaque growth, thereby mitigating lumen narrowing.^14^ Consistently, angiographic studies of patients with acute myocardial infarction have demonstrated that atherosclerotic lesions leading to occlusive luminal thrombosis after plaque rupture often do not cause significant luminal obstruction.^15^

In this review, we encompass the definition of coronary VP across available invasive and noninvasive diagnostic techniques and offer a comprehensive overview of current pharmacological and interventional preventive strategies for VP stabilization. Special emphasis is placed on identifying key research gaps for the development of effective preventive strategies aimed at reducing VP-related events, thereby improving outcomes in patients with coronary artery disease.

## VULNERABLE PLAQUE Destabilization

**Figure 1.**
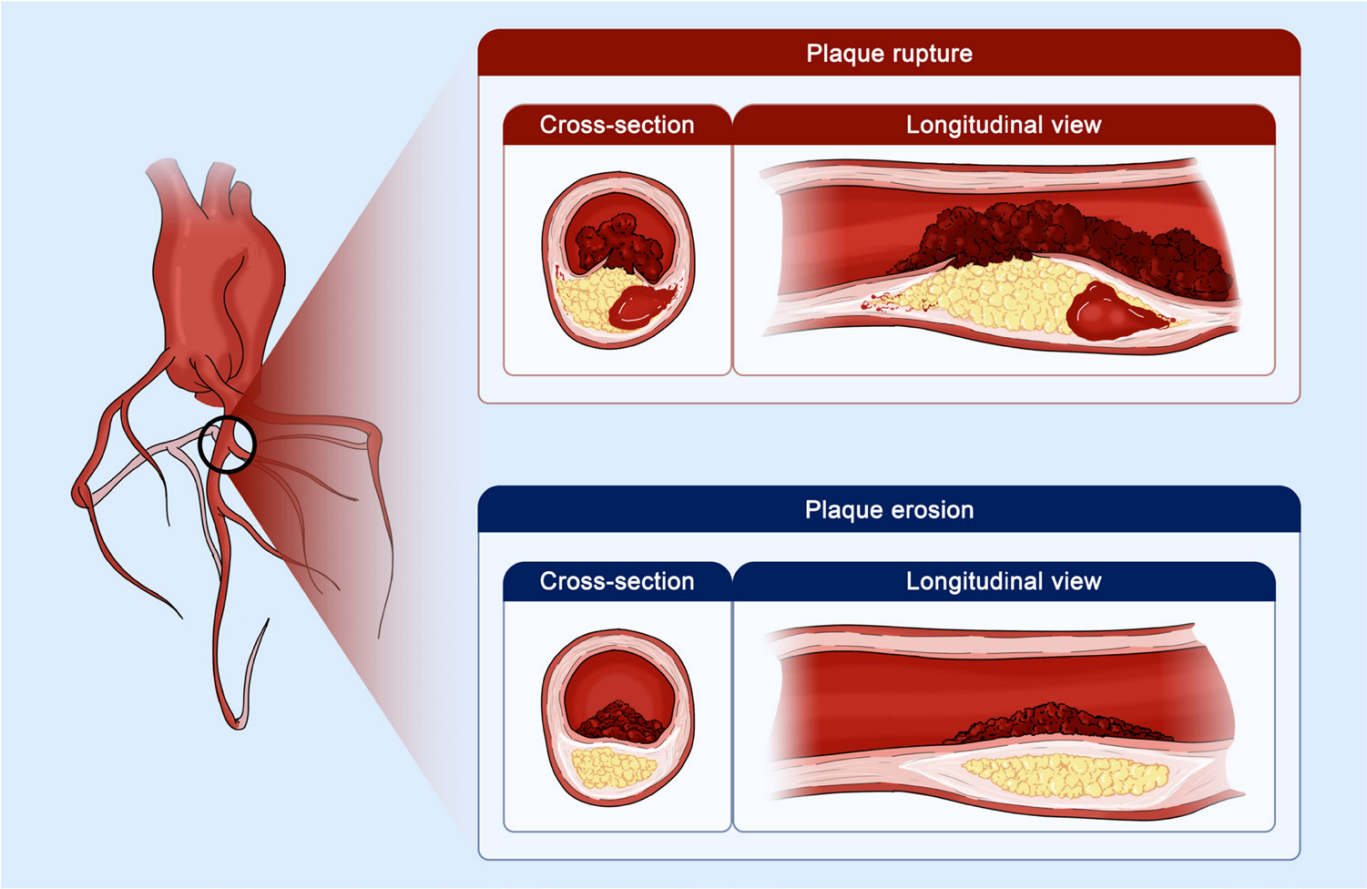
**Main progression pathways of destabilized vulnerable plaques (VPs).** High-risk characteristics of VPs predispose them to rupture or erosion. Ruptured plaques typically exhibit a thin fibrous cap and a large lipidic pool with a necrotic core and intraplaque hemorrhage. Rupture exposes the highly thrombogenic necrotic core material to the bloodstream thereby triggering thrombosis. In contrast, eroded plaques exhibit focal endothelial denudation, often in the context of plaques with a thick fibrous cap and without a large atheroma. This endothelial rarefaction exposes the underlying smooth muscle cells and extracellular matrix to the bloodstream, ultimately leading to thrombosis.

Autopsy, histopathology, and imaging studies have delineated the histopathology hallmarks of VP evolving into ruptured or eroded plaques (Figure 1).^16^ The susceptibility of plaques to rupture seems to depend on several factors, including the balance between smooth muscle cell-dependent production and inflammation-driven metalloproteinase-promoted breakdown of the extracellular matrix, plaque expansion, eccentricity, and angulation in relation to cap fatigue, endoluminal shear stress from blood flow, vasospasm, and microcalcifications.^6,9,17,18^ The fibrous cap, a tissue layer including densely woven type I and III collagen separating the plaque from the vessel lumen, is normally capable of bearing considerable tensile stress without breaking.^6^ The thin-cap fibroatheroma (TCFA) is considered a precursor of ruptured plaques, and histopathologic data have indicated that up to 95% of ruptures occur in plaques with fibrous caps <65 µm, infiltrated by inflammatory cells, predominantly macrophages, and few or absent smooth muscle cells.^6,9,17,18^ Nevertheless, cap thickness alone is deemed insufficient to precipitate plaque rupture, as not all plaques that disrupt have a thin fibrous cap and ruptured plaques with thin fibrous caps show other high-risk features, such as a large lipid pool with a highly thrombogenic necrotic core surrounded by foam cells and T cells, which synergistically contribute to the process.^6,9,16–18^ Lipid pools evolve into highly thrombogenic necrotic cores—around 6× more thrombogenic than other plaque components—through macrophage infiltration and lipid phagocytosis, which induce apoptosis, release acellular debris, and deposit free cholesterol crystals.^6,9,16,17^ In addition, neovascularization may contribute to the development of plaque hemorrhage with consequent rapid plaque expansion.^19^

Eroded plaques seem to be 2 to 3× less common than ruptured plaques.^16^ These lesions are characterized by an intact fibrous cap and focal discontinuity of an endothelial layer primarily composed of smooth muscle cells and a proteoglycan-rich matrix, a lipidic core (with limited or absent necrotic component), and sparse infiltrating macrophages and T cells.^16^ The thrombogenic substrate is less evident compared with plaque rupture, the fibrous cap is generally thicker, and the lipid pool tends to be smaller.^16,20^ Multiple concurrent factors, including extracellular matrix abnormalities, tissue factors, endothelial cell apoptosis, neutrophil extracellular trap formation, disturbed endothelial shear stress, and prothrombotic hematic conditions seem to be involved in the development of endothelial denudation and thrombosis.^16^ Although some studies report more favorable outcomes compared with ruptured plaques, eroded plaques have also been associated with thrombosis and sudden death.^16^

Neoatherosclerosis, a leading cause of in-stent restenosis and target lesion failure at long-term follow-up, has been defined as the presence of lipid-laden foamy macrophages within the neointimal tissue in the stented segment, with or without necrotic core, thin cap, and calcifications.^21,22^ Neoatherosclerotic TCFA rupture is a major cause of late stent thrombosis with mechanisms similar to those described for de novo coronary artery disease.^22^

## VULNERABLE PLAQUE Detection

Coronary angiography identifies deep plaque ulcerations and eroded plaque-related thrombosis with high specificity and low sensitivity. In fact, it is incapable of detecting occult lesions in apparently normal or mildly diseased vessel segments and cannot identify VPs, as it portrays foreshortened, bidimensional views of the lumen silhouette rather than directly imaging the diseased vessel wall.^23^

A wide array of invasive and noninvasive imaging techniques provide information on plaque burden, composition, and key histopathologic features of vulnerability, each with inherent advantages and disadvantages.^24^ Invasive coronary imaging techniques, namely intravascular ultrasound (IVUS), optical coherence tomography (OCT), and near-infrared spectroscopy (NIRS), have shown high correlation with histology and have been extensively used for the identification of VPs and the guidance of percutaneous coronary intervention (PCI).^24,25^ Each of these methods has unique merits and drawbacks.^24^ IVUS enables precise measurements of plaque burden and lumen obstruction, while its resolution is insufficient for detecting thin fibrous caps. The qualitative assessment of plaque composition according to tissue echogenicity with IVUS requires postprocessing through validated algorithms.^26^ OCT offers superior resolution for assessing plaque structure; in fact, it differentiates lipid from fibrous tissue accurately, enables the direct measurement of cap thickness, and quantifies calcification better than IVUS. It also properly identifies plaque erosion or rupture and plaque microstructures.^24,27,28^ However, OCT has limited penetration and requires contrast media administration. NIRS complements these modalities by detecting lipid-rich plaques with high accuracy but lacks detailed structural information, which limits its standalone diagnostic utility.^29–31^ A substantial body of evidence indicates that the identification of certain anatomic features—particularly TCFA (<65–75 µm), large lipid arc (>180°), minimal lumen area (MLA) <3.5 mm^2^, plaque burden ≥70%, macrophage infiltration, and lipid-core burden, whether individually or, ideally, in combination—is capable of pinpointing plaques at high risk of rupture, leading to adverse cardiovascular events, including ACSs and death.^30,32–34^ Integrating multiple diagnostic techniques can further enhance predictive accuracy by leveraging the strengths of each method’s discriminative capacity.^24^ Overall, while these methods carry only a minor risk of vessel injury and procedure-related complications, they are generally not suited for systematic screening—particularly in patients without an indication for coronary angiography.

Noninvasive imaging techniques, including coronary computed tomography angiography (CCTA), magnetic resonance imaging, and positron emission tomography, offer the potential for broader screening to identify patients requiring an invasive assessment or to guide treatment decisions.^35,36^ CCTA is widely adopted for the assessment of coronary artery disease and offers relatively high-resolution plaque characterization.^24,35^ Particular plaque characteristics at CCTA, like positive remodeling, low attenuation, spotty calcification, and the napkin-ring sign exhibited important correlations with adverse outcomes in large studies, and experts in the CAD-RADS 2.0 consensus suggested defining VP in the presence of at least 2 of these features.^37^ Magnetic resonance imaging excels in plaque quantification and composition analysis but is unlikely to provide sufficient resolution to accurately identify features like thin-capped coronary fibroatheroma.^38^ Moreover, it faces challenges with coronary artery imaging due to cardiac motion, limited sensitivity, and time-consuming protocols.^38^ Positron emission tomography, while promising for detecting metabolic plaque activity (macrophage activation, microcalcifications), requires further validation to establish its comparative prognostic value and integration in routine practice.^39^ In all, noninvasive techniques have lower resolution and specificity than intravascular imaging in the identification of specific VP high-risk features.^24^

**Figure 2.**
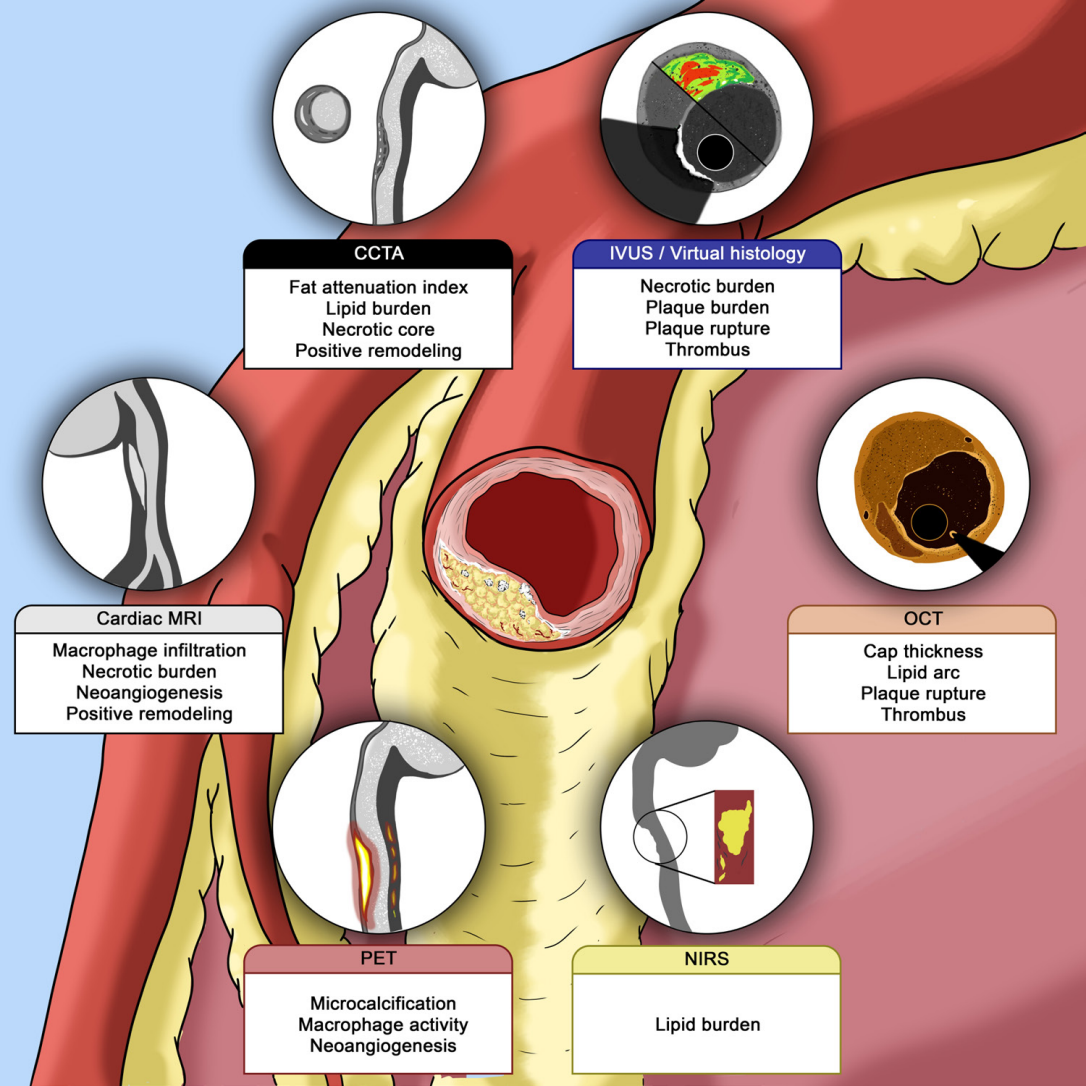
**Characteristics of vulnerable plaques (VPs) by different imaging modalities.** The figure summarizes the key features of VPs that can be identified by currently available invasive and noninvasive imaging techniques. A detailed discussion of each modality’s capabilities and limitations is provided in the Supplemental Material. CCTA indicates coronary computed tomography angiography; IVUS, intravascular ultrasound; MRI, magnetic resonance imaging; NIRS, near-infrared spectroscopy; OCT, optical coherence tomography; and PET, positron emission tomography.

Tables 1 and 2, along with Figure 2, summarize imaging-defined features of plaque vulnerability and their association with long-term clinical outcomes based on invasive and noninvasive assessments. A detailed analysis of VP detection methods, their mechanisms, and their prognostic capabilities is available in the Supplemental Material.

**Table 1.**
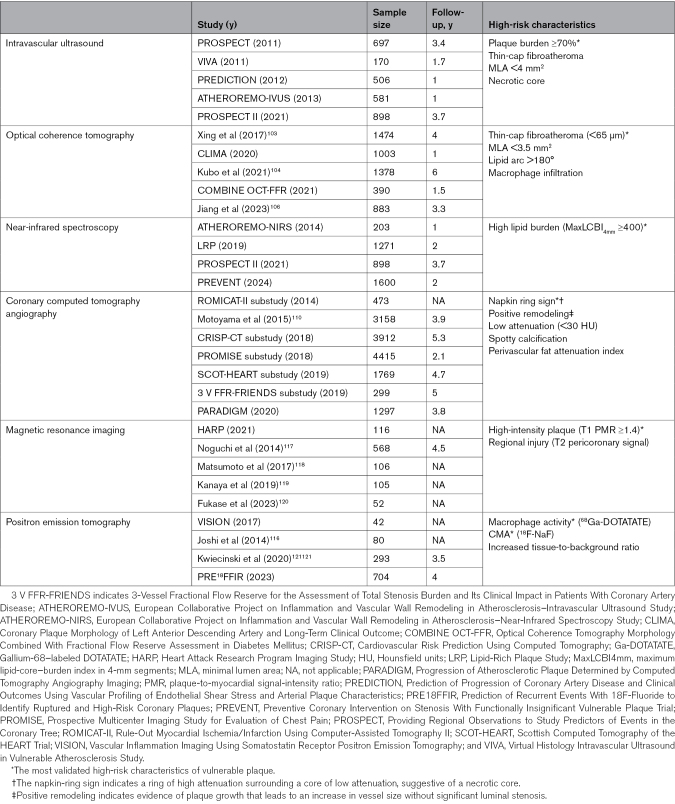
Characteristics of Vulnerable Plaques by Different Imaging Modalities

**Table 2.**
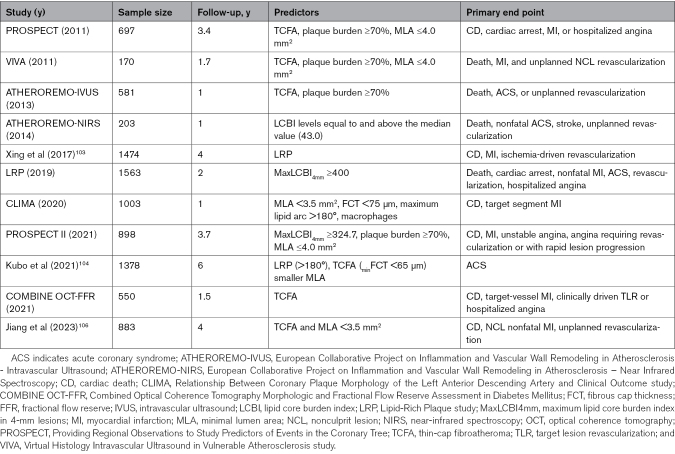
Studies Proving the Association Between the Characteristics of Untreated Vulnerable Coronary Plaques and Long-Term Clinical Outcomes

## Treatment of VULNERABLE PLAQUES

After several decades of investigations focusing on the identification of high-risk morphological features, the notion that single mechanisms and direct causes produce VP destabilization has been surpassed.^17^ A substantial body of evidence on systemic, modifiable or nonmodifiable, conventional or nonconventional risk factors influencing plaque stability has guided therapeutic efforts toward systemic strategies aimed at preventing coronary artery disease progression.^12^ VP development and rupture result from the complex interplay among traditional ischemic risk factors, coronary anatomic settings, individual functional responses, rheological effects, and genetic, hemodynamic, hormonal, dietary, and environmental conditions.^12^ The magnitude of the thrombotic reaction triggered by VP destabilization is essential in determining the clinical relevance of the event.^18^ Understanding these complexities is essential to explain the limited discriminative value of current predictive models for VP rupture and the use of effective local and systemic preventive strategies.

### Medical Therapy

While VP detection relies on an instantaneous assessment, the dynamic nature of coronary artery disease implies that plaques without high-risk features can later develop characteristics of vulnerability. VPs essentially reflect systemic disorders, and ruptured plaques causing ACS coexist with complex rupture-prone plaques at another coronary segment in ≈40% to 50% of patients.^40^ In this context, medical therapy targeting the entire coronary tree stabilizes VPs by promoting the transition of their structural components from high to low rupture risk. Intravascular imaging of coronary plaques may be instrumental in guiding pharmacotherapy and justifying the adoption of more potent lipid-lowering medications and intensified preventive antithrombotic regimens.

#### Lipid-Lowering

**Figure 3.**
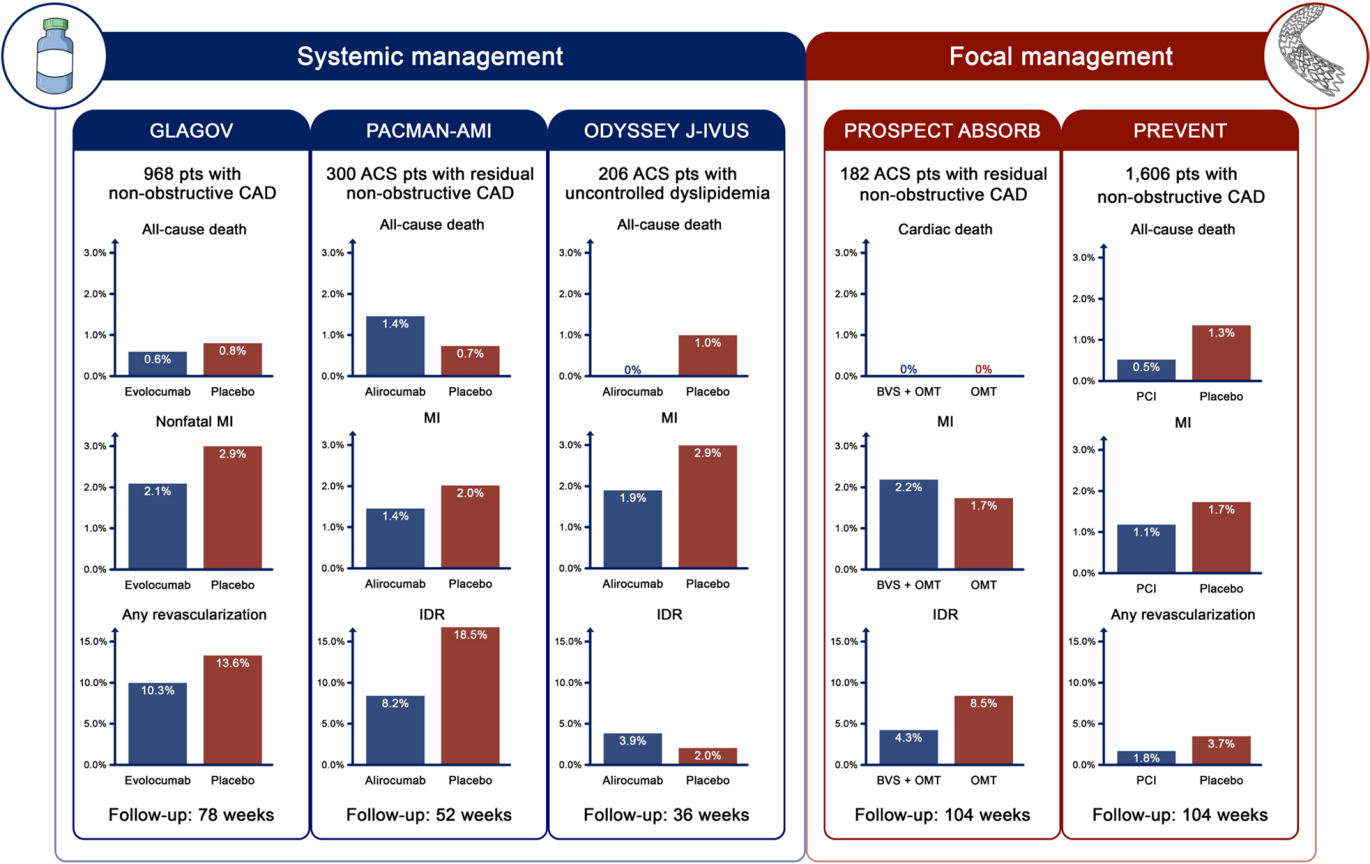
**Outcomes of systemic and focal invasive management of vulnerable plaques (VPs).** Robust evidence from several large-scale randomized trials supports the efficacy of lipid-lowering medications in improving long-term cardiovascular outcomes. Although the plaque-modifying effects of statins and PCSK9 inhibitors have been directly and mechanistically evaluated in a limited number of imaging-based studies, these medications have generally shown to decrease plaque volume, mitigate coronary artery disease progression, and stabilize plaque composition with a transition of VPs from high- to low-risk features and a reduced development of new VPs. In parallel, the focal treatment of selected, isolated, nonobstructive VPs has emerged as a promising preventive strategy. Clinical trials and advancements in device performance and biocompatibility have yielded encouraging results, particularly when specific intravascular imaging criteria such as an minimal lumen area (MLA) <4.00 mm^2^, a plaque burden >70%, and the presence of thin-cap fibroatheroma are met. However, this approach is tempered by the invasiveness of the procedure and the need for a permanent metallic implant. Evaluating the balance between the benefits and limitations of these strategies represents a pivotal objective of ongoing and upcoming investigations. ACS indicates acute coronary syndrome; BVS, bioresorbable vascular scaffold; CAD, coronary artery disease; IDR, ischemia-driven revascularization; MI, myocardial infarction; OMT, optimal medical therapy; and PCI, percutaneous coronary intervention.

A substantial body of evidence indicates that lipid-lowering medications reduce plaque volume and stabilize plaque composition, lowering myocardial infarction, hospitalization for unstable angina, revascularization, and stroke at long-term follow-up.^41^ Although most of the events are plausibly linked to plaque destabilization, the plaque-modifying implications of statin treatment were directly assessed in a limited number of intravascular imaging-based investigations (Table S1; Figure 3).

The ASTEROID (A Study to Evaluate the Effect of Rosuvastatin on Intravascular Ultrasound-Derived Atheroma Regression) and IBIS-4 (The Integrated Biomarkers and Imaging Study 4) single-arm IVUS studies, showed significantly reduced atheroma volumes at long-term follow-up compared with baseline in patients treated with high-dose rosuvastatin.^42,43^ The randomized YELLOW trial (Reduction in Yellow Plaque by Aggressive Lipid Lowering Therapy) showed by NIRS-IVUS that after 7 weeks rosuvastatin high-dose was associated with atheroma volume reduction, and Maximum Lipid Core Burden Index within a 4 mm segment decreased compared with standard of care lipid-lowering therapy.^44^ These effects were generally dose-dependent and unrelated to the type of statin. In the EASY-FIT trial (Effect of Atorvastatin Therapy on Fibrous-Cap Thickness), testing different atorvastatin doses (20 mg versus 5 mg), the higher-intensity regimen significantly improved fibrous cap thickness and reduced macrophage infiltration, as assessed by OCT.^45^ In the 20 mg/d atorvastatin group, all 6 TCFAs detected at baseline OCT evolved into thick-cap fibroatheroma at follow-up, while in the 5 mg/d atorvastatin group, only 2 of 6 lesions evolved into thick-cap fibroatheroma.^45^ Consistently, the REVERSAL randomized trial (Reversal of Atherosclerosis With Statins in Individuals With Aggressive Lipid-Lowering) demonstrated in 654 patients that high-dose atorvastatin (80 mg) significantly reduced 18-month percent atheroma volume compared with moderate-dose pravastatin (40 mg).^46^ In contrast, in the SATURN trial (Study of Coronary Atheroma by Intravascular Ultrasound: Rosuvastatin Versus Atorvastatin), the comparison between high-dose rosuvastatin and high-dose atorvastatin in 1039 patients showed no significant difference in IVUS-assessed percent atheroma volume reduction, though rosuvastatin showed an advantage in terms of normalized total atheroma volume.^47^

Further studies suggest that adding lipid-lowering agents on top of statins may further enhance the benefits.^48^ The PCSK9 (Proprotein Convertase Subtilisin/Kexin Type 9) inhibitors evolocumab and alirocumab showed significant plaque volume reductions against placebo as assessed by intravascular imaging.^49–51^ In the multicenter, double-blind, placebo-controlled GLAGOV trial (Global Assessment of Plaque Regression With a PCSK9 Antibody as Measured by Intravascular Ultrasound), including 846 patients with baseline and follow-up IVUS assessment, monthly subcutaneous injection of evolocumab (420 mg), reduced after 78 weeks percent plaque volume (−1.0% [95% CI, −1.8 to −0.6]) and normalized total atheroma volume (−4.9 mm^3^ [95% CI, −7.3 to −2.5]) compared with placebo. Plaque regression was more frequent in patients assigned to evolocumab than in those assigned to placebo.^49^ Consistently, in the HUYGENS trial (High-Resolution Underlying-Plaque Assessment With Evolocumab Using Intravascular Ultrasound), evolocumab demonstrated an increased minimum fibrous cap thickness (42.7 versus 21.5 µm; *P*=0.015) and a reduced lipid arc extension (−57.5% versus −31.4%; *P*=0.04) compared with placebo at 52-week OCT.^50^ Later, the double-blind, placebo-controlled, randomized PACMAN-AMI trial (Effects of the PCSK9 Antibody Alirocumab on Coronary Atherosclerosis in Patients With Acute Myocardial Infarction) showed that adding biweekly subcutaneous alirocumab (150 mg) to high-dose statin was associated at 52-week NIRS-IVUS assessment with improved mean reductions in percent atheroma volume (−1.21% [95% CI, −1.78 to −0.65]) and maxLBCI_4mm_ (−41.24 [95% CI, −70.71 to 11.77]) and a mean increase in minimal fibrous cap thickness (29.65 µm [95% CI, 11.75–47.55]) compared with placebo.^51^ In this context, the ODYSSEY-J IVUS trial (Effect of Alirocumab on Coronary Atheroma Volume in Japanese Patients With Acute Coronary Syndrome) may be an outlier as it did not show benefits in normalized total atheroma volume associated with alirocumab compared with placebo.^52^ Finally, the PRECISE-IVUS (Plaque Regression With Cholesterol Absorption Inhibitor or Synthesis Inhibitor Evaluated by Intravascular Ultrasound) and EVAPORATE (Effect of Vascepa on Improving Coronary Atherosclerosis in People With Elevated Triglycerides Taking Statin Therapy) trials indicated that combining statins with ezetimibe or icosapent ethyl may further enhance plaque regression, while there is no information on the effects of novel drugs targeting lipoprotein(a) on plaque destabilization.^53–55^

#### Anti-Inflammatory

Imaging-based studies assessing the effects of colchicine on VPs have shown mixed results.^56,57^ The randomized, double-blind, COLOCT trial (Colchicine on Coronary Plaque Stability Trial Assessed by Optical Coherence Tomography), including 128 patients with ACS and lipid pool arc >90°, showed at the 12-month OCT follow-up significantly increased minimal fibrous cap thickness (34.2 µm [95% CI, 9.7–58.6]), reduced average lipid arc (−10.5° [95% CI, −17.7 to −3.4]), and decreased mean angular extension of macrophages (−6.0° [95% CI, −11.8 to −0.2]) after colchicine compared with placebo.^58^ In contrast, in the double-blind, placebo-controlled COCOMO-ACS trial (Colchicine for Coronary Plaque Modification in Acute Coronary Syndrome), including 64 patients with non–ST-segment–elevation myocardial infarction, at a median of 17.8 months, no significant improvement in minimum fibrous cap thickness and maximum lipid arc in OCT-imaged nonculprit lesions was shown.^56^

#### Antithrombotic

Although antithrombotic medications do not alter plaque composition or prevent rupture, some large-scale randomized trials of patients with established coronary artery disease, diabetes, prior myocardial infarction, and prior coronary revascularization, have indicated significant reductions in major adverse cardiovascular events (MACE) and generally myocardial infarction, in patients assigned to prolonged intensified antithrombotic treatments compared with placebo.^59–61^ However, these benefits invariably occurred at the cost of an increased risk of major bleeding, indicating that these regimens are justifiable only in selective settings.^59,62^ Finally, recent evidence showed that, in patients who had undergone PCI and completed the recommended initial dual antiplatelet therapy period, P2Y_12_ inhibitor monotherapy is associated with reduced incidences of MACE, especially myocardial infarction, and a similar occurrence of major bleeding compared with aspirin monotherapy.^63–65^

### Interventional Treatment

In this context, preventive stenting constitutes a paradigm shift in which PCI is not performed to treat an ACS or a significant stenosis but to seal VPs before rupture or erosion. The hypothesis is that the stent constitutes a physical barrier ultimately stabilizing the VP by excluding its structures from the lumen and inducing neointimal hyperplasia.^66,67^ However, this strategy raises concerns about the risk of acute distal embolization of plaque material, periprocedural myocardial infarction as well as higher rates of target lesion failure due to in-stent restenosis and stent thrombosis.^31,68,69^

In the PROSPECT-ABSORB trial (Providing Regional Observations to Study Predictors of Events in the Coronary Tree - ABSORB), 182 selected patients with recent myocardial infarction and successful PCI of culprit disease were randomized to preventive bioresorbable vascular scaffold implantation plus optimal medical therapy or optimal medical therapy alone for the treatment of NIRS-IVUS-defined nonobstructive nonculprit VPs.^70^ At the 2 year NIRS-IVUS follow-up, MLA was superior in the bioresorbable scaffold group compared with the medical therapy alone group (least square mean difference, 3.9 mm^2^ [95% CI, 3.3–4.5]).^70^ At 4 years, a broad composite of MACE was not significantly different between groups (4.3% versus 10.7%; odds ratio, 0.38 [95% CI, 0.11–1.30]).^70^

More recently, the PREVENT trial (Preventive PCI of Vulnerable Plaques Trial) randomly assigned 1606 patients with predominantly chronic coronary syndrome requiring PCI to preventive stenting of nonculprit FFR negative (>0.80) disease with at least 2 high-risk characteristics at intravascular imaging plus optimal medical therapy (n=780) or optimal medical therapy alone (n=776).^71^ The qualifying plaque characteristics resembled the definition emerged from the PROSPECT II study (MLA <4.0 mm^2^ by IVUS or OCT; a plaque burden of >70% by IVUS; a lipid-rich plaque by NIRS) plus thin-cap fibroatheroma detected by radiofrequency IVUS or OCT. The median number of VPs per patient was 1 (interquartile range, 1-1] with a mean percentage diameter stenosis of 56.5%±9.2% and a mean length of 23.6 mm±9.2 mm in patients assigned to preventive PCI.^71^ IVUS was the most used intravascular imaging technique (95%), and the most represented VP qualifying characteristics were an MLA <4.00 mm^2^ (97%) and a plaque burden >70% (97%).^71^ Of note, preventive stenting-related PCI complications, including acute thrombosis, major distal dissection, distal embolization, side branch occlusion, and coronary perforation, were extremely low (<1%).^71^ At 2 years, the primary composite end point of cardiac death, target-vessel myocardial infarction, ischemia-driven target-vessel revascularization, or hospitalization due to unstable or progressive angina was significantly lower in the PCI group compared with the optimal medical therapy group (0.4% versus 3.4%; −3.0% [95% CI, −4.4 to −1.8]).^71^ The difference was driven by hospitalization for unstable or progressive angina (0.1% versus 1.5%) and ischemia-driven target lesion revascularization (0.1% versus 2.4%; −2.3% [95% CI, −3.4 to −1.2]).^71^ At the maximum available follow-up of 7 years, the difference between treatments persisted, though the trial was underpowered to draw definitive conclusions on long-term outcomes.^71^ However, hospitalization due to unstable or progressive angina (−0.7% versus 4.9%; −4.2 [95% CI, −7.2 to −1.4]) remained significantly lower in the interventional group compared with the medical therapy alone group.^71^

## Critical Reading and Future Directions

### Vulnerable Plaque Detection

Intravascular imaging is an established modality for identifying VPs, yet each technique has inherent limitations in detecting the full spectrum of high-risk morphological features. As such, the use of a single imaging tool may not provide a comprehensive risk profile. Advancing in imaging catheter technology (eg, high-definition IVUS systems) and combining multiple imaging techniques (eg, IVUS-OCT, OCT-NIRS, OCT-near-infrared autofluorescence) may significantly improve the positive predictive value.^72–74^

Adding to the importance of anatomic assessment, emerging evidence highlights the potential of incorporating hemodynamic plaque and vessel characteristics to better estimate the risk of plaque-related adverse events. A small study using fractional flow reserve (FFR) derived from CCTA pioneered this field, demonstrating that the combination of adverse plaque morphology and unfavorable hemodynamics significantly improved the discrimination of ACS compared with either factor alone, achieving an area under the curve of 0.789.^75^ This concept was further expanded in the EMERALD-II study (Exploring the Mechanism of Plaque Rupture in Acute Coronary Syndrome Using Coronary CT Angiography and Computational Fluid Dynamics II), where, across 2088 nonculprit and 363 culprit lesions, an artificial intelligence-enabled quantitative coronary plaque and hemodynamic analysis using the same method, identified the variation of FFR across the lesion (ΔFFR) as the strongest predictor of ACS, followed by wall shear stress and known anatomic plaque characteristics.^76^ Notably, when atherosclerotic distribution patterns (focal versus diffuse) were assessed using FFR pullback gradients, focal stenoses resulted associated with greater plaque burden, predominantly lipid-rich composition, and a higher prevalence of TCFA, whereas diffuse atherosclerosis showed a greater prevalence of calcification—highlighting the potential influence of local hemodynamics on plaque phenotype and, ultimately, vulnerability.^77,78^ Building on this, a sub-analysis of the PROSPECT II study, which included 319 angiographically intermediate lesions with matched IVUS/NIRS and FFR/instantaneous wave-free ratio measurements, showed that physiologically significant lesions more frequently exhibited high-risk anatomic features and were independently associated with unfavorable characteristics. Thus, the development and validation of prediction tools that integrate advanced anatomic and physiological plaque metrics may represent the next critical frontier in the field, and a prospective natural history study of coronary atherosclerosis using next-generation imaging and physiological techniques is warranted.

Despite these advances, identifying apparently healthy individuals who are at high risk for developing an ACS and require intensified prevention remains a significant challenge, and individual plaque features may have insufficient sensitivity for this approach. For example, in the PROSPECT II study, regardless of multivariable adjustment, patients with diabetes more frequently experienced nonculprit lesion-related spontaneous myocardial infarction, despite similar VP distribution between groups.^79^ Also, coronary lesions without physiological significance demonstrated a moderate-to-high prevalence of high-risk plaque features, which may help explain the residual risk associated with conservative, noninterventional management.^80^ Moreover, most of the studies addressing the prediction of VP rupture used univariate regression or restricted multivariable models due to limitations in the number of patients and lesions.

These insights underscore the likelihood that systemic factors may modulate plaque behavior in ways that are not fully captured to date—highlighting the need to improve risk stratification strategies to better identify individuals who may benefit from more intensive preventive care. Advances in proteomics, the entire set of proteins produced or modified by an organism, and radiomics, the spectrum of quantitative metrics included in imaging data, combined with biometric, demographic, clinical, and genetic information, may improve, through artificial intelligence, the earlier and more accurate detection of VPs.^81,82^

### Medical Therapy

Patients with multiple VPs may require more aggressive systemic treatment with lipid-lowering and anti-inflammatory medications, in addition to lifestyle modifications and strict risk factor control.^83^ Moreover, in patients with a high ischemic risk profile and recurrent ACS due to VP rupture, despite achieving optimal medical therapy, intensifying antithrombotic regimens may be justified, provided the expected bleeding risk does not outweigh the potential benefits in preventing ischemic events, unless novel antithrombotic compounds demonstrating minimal or absent bleeding risk are proven efficacious.^84,85^

While numerous studies have tested different lipid-lowering medications, the armamentarium against inflammation is essentially limited to colchicine and shows controversial results.^4^ Indeed, previous improvements in MACE associated with colchicine in pivotal trials were recently questioned by the large-scale placebo-controlled CLEAR SYNERGY OASIS 9 trial (Colchicine and Spironolactone in Patients With Myocardial Infarction / SYNERGY Stent Registry—Organization to Assess Strategies for Ischemic Syndromes 9).^86^ In this context, the results of trials testing novel anti-inflammatory treatments, such as the interleukin-6 inhibitor ziltivekimab for secondary prevention of patients with recent myocardial infarction, in the international, randomized, double-blind, placebo-controlled ARTEMIS trial (Effects of Ziltivekimab Versus Placebo on Cardiovascular Outcomes in Patients With Acute Myocardial Infarction; URL: https:// www.clinicaltrials.gov; Unique identifier: NCT06118281), are awaited. Moreover, emerging glucose-lowering agents with hypothesized pleiotropic effects may contribute to plaque involution. Sodium-glucose cotransporter-2 inhibitors have shown class-effect signals in improving morphological features of plaque vulnerability in early noninvasive and invasive imaging studies; however, these findings are limited by their observational nature.^87^ Concurrently, randomized clinical trial data on glucagon-like peptide-1 receptor agonists are accumulating, demonstrating cardiovascular benefits, particularly in reducing myocardial infarction.^88^ The role of these novel compounds in plaque modification warrants dedicated investigation.

### Preventive PCI

Regardless of high-risk morphological features, the interventional treatment of high-grade vulnerable coronary stenoses is primarily driven by objective evidence of reduced coronary perfusion.^4^ In contrast, nonobstructive VPs hold the potential to rupture but do not cause myocardial ischemia.^89^ Available data indicate that, within 1 year of invasive assessment, up to 3-quarters of plaques stabilize after cycles of rupture and repair.^89^ These findings highlight that substantial proportions of VPs treated by preventive stenting may ultimatel not disrupt over time.^89^ Nevertheless, treating selected, isolated nonobstructive VPs of limited extent may be a successful preventive strategy, especially when PREVENT-like high-risk features are satisfied.^71^

However, preventive stenting seems to be unreasonable for multiple VPs, especially when associated with mild lumen obstruction. In the PREVENT trial, an overly restrictive patient selection may have occurred, as only 28.5% of 5627 patients screened for eligibility were included over a 6-year accrual period.^71^ Against this background, it is important to note that in PREVENT, 2- and 7-year primary end point incidences were several times lower than target lesion failure and MACE incidences in previous studies with second-generation drug-eluting stents, likely reflecting the treatment of only short and noncomplex VPs.^71,90–93^ These statements are also supported by the original assumptions of the trial, according to which the expected 2-year incidence of the primary outcome (8.5%) was >20× higher than that observed in the interventional group (0.4%), making PREVENT significantly underpowered.^71^ In addition, the predominant inclusion of target lesions with an MLA <4.0 mm^2^, frequently corresponding to angiographic diameter stenoses approaching the threshold of significant coronary artery disease, may have determined the predominant inclusion of VPs with a higher likelihood of causing myocardial ischemia without acute myocardial infarction, and undergoing stenting for symptoms unrelated to plaque rupture. In addition, in the context of an open-label design, the subjective perception and physician’s interpretation of angina and ischemic symptoms along with the awareness of untreated high-risk plaques may artifactually increase the rate of unplanned revascularization in the noninvasive control group.^94^ These statements are supported by the predominance of unstable or progressive angina and ischemia-driven target-vessel revascularization in the trial.^71^

Although in PREVENT the rates of periprocedural complications and long-term stenting-related complications were excellent, with no stent thrombosis associated with preventive drug-eluting stent implantation, it remains unclear whether liberal preventive stenting of more complex and diffuse vulnerable lesions would increase the rates of in-stent restenosis and stent thrombosis.^71^ PROSPECT-ABSORB and, partially (≈33%), PREVENT opted for bioabsorbable vascular scaffolds rather than drug-eluting stents to avoid the long-term issues associated with permanent metallic layers for nonobstructive VPs.^70,71^ For similar reasons, drug-coated balloon angioplasty may be an appealing treatment option.^95,96^ Explorative studies have shown that drug-coated balloons may reduce atheroma volume and increase fibrous cap thickness, but no randomized trials assessing their comparative efficacy and safety versus optimal medical therapy or preventive stenting for VPs exist to date.^97^ In this regard, natural history studies such as PROSPECT and VIVA (Virtual Histology-Intravascular Ultrasound in Vulnerable Atherosclerosis Study), showed that the incidence of culprit-lesion-related MACE was similar to or higher than that of nonobstructive nonculprit lesions (VPs) treated by medical therapy.^33,98^ However, PROSPECT II differed from PROSPECT and VIVA by displaying a higher incidence of events related to nonculprit than culprit lesions.^30^

Ongoing trials will fuel the discussion surrounding preventive stenting for VPs. The VULNERABLE trial (Treatment of Functionally Non-Significant Vulnerable Plaques in Patients With Multivessel ST-Elevation Myocardial Infarction; URL: https://www.clinicaltrials.gov; Unique identifier: NCT05599061) is randomly allocating 600 patients admitted for ST-segment–elevation myocardial infarction patients with nonobstructive (40–69% by angiography) nonculprit FFR negative (>0.80) lesions with OCT-defined VP features to drug-eluting stent-based PCI plus optimal medical therapy or optimal medical therapy. The trial will assess the primary composite outcome of target-vessel failure between groups at a follow-up of 4 years. Similarly, The Randomized Controlled Study on the Effectiveness of Interventional Therapy for Non-Flow-Limiting Vulnerable Plaques (URL: https://www.clinicaltrials.gov; Unique identifier: NCT06855537) will randomly assign 2190 ACS with functionally non-significant stenoses and OCT-defined features of vulnerability to preventive PCI plus optimal medical therapy or optimal medical therapy alone, for a 2-year composite clinical end point. The COMBINE-INTERVENE trial (Combined Ischemia- and Vulnerable-Plaque–Guided Intervention Trial; URL: https://www.clinicaltrials.gov; Unique identifier: NCT05333068) will provide data on the 2-year outcomes of >1200 patients randomly assigned to PCI of OCT-defined VPs, ruptured, or eroded plaques with FFR <0.75, or PCI of plaques not assessed by OCT with FFR <0.80. Differently, the ongoing INTERCLIMA trial (Interventional Strategy for Non-Culprit Lesions With Major Vulnerability Criteria Identified by Optical Coherence Tomography in ACS; URL: https://www.clinicaltrials.gov; Unique identifier: NCT05027984) will randomly assign 1420 patients with 40% to 70% coronary lesions assessed by angiography to OCT-guided PCI based on the detection of key high rupture-risk characteristics or functionally guided (FFR, instantaneous wave-free ratio, or rest full-cycle ratio) PCI, essentially based on the hemodynamic significance (Table 3). Alternative interventional strategies for treating VPs that intend to minimize stent-related adverse effects for treating VPs are under investigation. Drug-coated balloons are currently being investigated for the treatment of nonculprit VPs identified by intravascular imaging in patients with ACS, in comparison with optimal medical therapy, in the RESTORE (Preventive Drug-Coated Balloon Angioplasty in Vulnerable Atherosclerotic Plaque Trial; URL: https://www.clinicaltrials.gov; Unique identifier: NCT06365502) and PASSIVATE-CAP (Passivation of Vulnerable Coronary Atherosclerotic Plaques; URL: https://www.clinicaltrials.gov; Unique identifier: NCT06416813) trials. Finally, the POLARSTAR trial (Polar Cryo-Energy Lesion Stabilization Research in Coronary Arteries Study Using Cryotherapy; URL: https://www.clinicaltrials.gov; Unique identifier: NCT05600088) is investigating intracoronary catheter-based cryotherapy to treat coronary plaques that are deemed at high risk of rupture by CCTA.

**Table 3.**
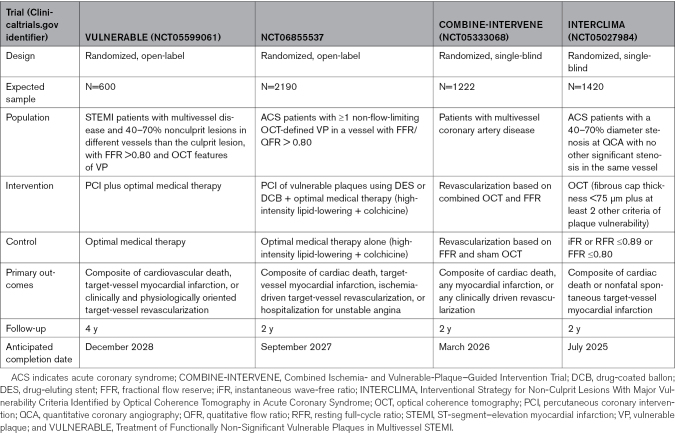
Ongoing Major Randomized Trials on the Invasive Management of the Vulnerable Coronary Plaque

## Conclusions

Advances in the detection and management of vulnerable coronary plaques hold promise for improving outcomes in patients with coronary artery disease. However, the positive predictive value of current invasive and noninvasive methods remains suboptimal, and further technological diagnostic improvements, especially by multimodality imaging catheter innovations and artificial intelligence support, may set new standards. In this framework, demographic, biometric, clinical, and genetic conditions must be incorporated into predictive models and considered in the development of preventive treatment strategies. Medical therapy targeting the entire coronary tree stabilizes VPs and reduces the development of unstable lesions, in association with lifestyle modifications and preventive antithrombotic therapy for high-risk and recurrent-event patients. Nevertheless, recent evidence also indicates that, in selected cases, intravascular imaging-guided preventive stenting provides effective plaque sealing and passivation.

## ARTICLE INFORMATION

### Sources of Funding

None.

### Disclosures

Dr Capodanno declares advisory board membership with Abbott Vascular, Amgen, Bristol-Myers Squibb, and Vectura; data monitoring committee roles with Amgen; and receipt of speakers’ honoraria from Chiesi, Daiichi Sankyo, Novo Nordisk, Menarini, Sanofi, and Terumo. The other authors report no conflicts.

### Supplemental Material

Supplemental Text

Table S1

References 99–116

## Supplementary Material
