# Supplementary material for: Advances in the Detection and Management of Vulnerable Coronary Plaques
Source: Circ Cardiovasc Interv. 2025 Jul 28;18(8):e015529. doi: 10.1161/CIRCINTERVENTIONS.125.015529 (PMC12356569; doi:10.1161/CIRCINTERVENTIONS.125.015529)
Supplement: Supplementary file 1 [file hcv-18-e015529-s001.pdf]

SUPPLEMENTAL MATERIAL

Advances in the Detection and Management of Vulnerable Coronary Plaques

Marco Spagnolo, MD, MSc;[a] * Daniele Giacoppo, MD, MSc, PhD;[a] * Claudio Laudani, MD, MSc;[a] Antonio Greco, MD, PhD;[a] Simone Finocchiaro, MD, MSc;[a] Maria Sara Mauro, MD, MSc;[a] Antonino Imbesi, MD, MSc;[a] Davide Capodanno, MD, PhD.[a]

[a] Division of Cardiology, Azienda Ospedaliero-Universitaria Policlinico, Policlinico "G. Rodolico–San Marco", University of Catania, Catania, Italy

* Equal contribution.

INDEX

Supplemental Text (page 2)

Supplemental Table (Table S1) (page 10)

SUPPLEMENTAL TEXT.

VULNERABLE PLAQUE DETECTION

The identification of VPs may be instrumental for preventing their destabilization. Although coronary angiography identifies deep plaque ulcerations and eroded plaque-related thrombosis with high specificity and low sensitivity, it is incapable of detecting occult lesions in apparently normal or mildly diseased vessel segments and cannot identify VPs as it portrays foreshortened bidimensional views of the lumen silhouette rather than directly imaging the disease vessel wall.[99, 100] A wide array of invasive and non-invasive imaging techniques provides information on plaque burden, composition, and key histopathological features of vulnerability with inherent advantages and disadvantages (**Table 1**, **Figure 2**).[12, 24]

Invasive coronary imaging techniques—intravascular ultrasound (IVUS), optical coherence tomography (OCT), and near-infrared spectroscopy (NIRS)—have shown high correlation with histology and have been extensively employed for the identification of VPs and the guidance of percutaneous coronary intervention (PCI), providing the most accurate in vivo definition of VPs.[100] However, these methods also carry a risk of vessel injury during image acquisition and, more in general, procedure-related complications, cannot be considered for systematic screening of patients, especially in those without an indication for coronary angiography, and depending on anatomic factors, may be technically incapable of inspecting distal segments and secondary coronary branches.

Non-invasive imaging techniques—coronary computed tomography angiography (CCTA), magnetic resonance imaging (MRI), and positron emission tomography (PET)—offer the potential for broader screening to identify patients requiring an invasive assessment or guide treatment decisions.[12, 24, 36] Nevertheless, non-invasive techniques have lower resolution and specificity than intravascular imaging in identifying specific VP high-risk features.[12, 24, 36]

Invasive Techniques

Intravascular Ultrasound

Gray-scale intravascular ultrasound (IVUS) requires mechanical-state rotating (30-45 MHz) or solid-state electronic multiple-phased arrays (~20 MHz) transducers mounted at the tip of the imaging catheter positioned across the coronary segment of interest that emit ultrasounds and provide cross-sectional images deriving from the conversion of acoustic waves backscattered off the coronary structures.[26] Gray-scale IVUS enables the definition of segmental plaque burden and geometry, provides precise measurements of the degree of luminal obstruction, identifies thrombotic material, and guides PCI.[26] However, the qualitative assessment of plaque composition according to tissue echogenicity requires post-processing through validated algorithms—virtual histology IVUS (VH-IVUS), integrated-backscatter IVUS, and iMAP—overlying on grey-scale images a color-coded map coding the plaque components.[26, 35] The axial resolution of IVUS (~150 μm) does not allow the direct measurement of the fibrous cap thickness and the definition of TCFA primarily relies on the absence of a visible fibrous cap along with the identification of a confluent necrotic core delimiting the lumen.[26]

The PROSPECT study, including 697 patients admitted for ACS who underwent successful PCI and systematic screening of three-vessel proximal residual non-culprit coronary artery disease by gray-scale and VH-IVUS, prospectively validated the association of TCFA (HR 3.35, 95% CI 1.77-6.36), plaque burden ≥70% (HR 5.03, 95% CI 2.51-10.11), and minimum lumen area (MLA) ≤4.00 mm^2 (HR 3.35, 95% CI 1.61-6.42) with major adverse cardiovascular events (MACE) over a median follow-up of 3.4 years, though the majority of events included unstable or progressive angina.[33] Despite these strong associations, IVUS-based screening sensitivity was modest as only 26 out of 51 (51.0%) non-culprit lesions associated with events were TCFAs, and of these, only 8 (30.8%) showed also a plaque burden ≥70% and an MLA <4.00 mm^2.[33] Although the incremental combination of high-risk features showed substantially improved predictive accuracy, only 1 in 6 lesions associated with MACE at follow-up showed TCFA, MLA <4.00 mm^2, and plaque burden ≥70%.[33] Consistent findings were observed in the VIVA study, including 170 patients referred for PCI and three-vessel VH-IVUS evaluation, in which TCFA (HR 8.16, 95% CI 1.78-37.32), plaque

burden >70% (HR 7.48, 95% CI 2.50-22.31), and MLA ≤4.00 mm^2 (HR 2.91, 95% CI 1.07-7.91) were associated with MACE a median follow-up of 625 days.[98] Later, the ATHEROREMO-IVUS study demonstrated in 581 patients undergoing PCI and VH-IVUS assessment the significant multivariable-adjusted association of TCFA (HR 1.98, 95% CI 1.09-3.60) and plaque burden >70% (HR 2.90, 95% CI 1.60-5.25) with 1-year non-culprit lesion-related MACE.[101] The positive predictive value improved only after combining individual risk characteristics.[101]

Optical Coherence Tomography

Frequency-domain OCT catheters emit near-infrared light with variable frequency ("swept-laser") through a rotating optical fiber coupled with an imaging lens.[28, 100] The interference pattern at different wavelengths (~1.25–1.35 μm) is processed by Fourier transformation to provide the amplitude profile and echo time delay of the light backscattered off the coronary structures.[28, 100] OCT axial resolution (10–15 μm) is about ∼10 times greater than that of IVUS, enabling an improved assessment of superficial plaque composition and microstructures.[28, 100] In comparison with IVUS, several studies have shown that OCT differentiates lipid from fibrous tissue accurately, quantifies calcium more properly, and provides direct measurement of cap thickness.[24, 28] However, OCT light has poor penetration depth (1.0–2.5 mm), approximately 4 times lower than IVUS (4.0-8.0 mm), and limited diffusion within the lipid pool and necrotic core, making this imaging technique less accurate than IVUS for measuring the atheroma depth and volume.[26, 35, 100] In addition, since red blood cells scatter the light, acquiring OCT images requires contrast media injection to clear the vessel from blood during high-speed motorized pullback.[24, 28]

In the CLIMA study, including 1,003 patients undergoing OCT examination of non-culprit proximal left anterior descending artery disease, the simultaneous observation of MLA <3.5 mm^2, TCFA with cap thickness <75 μm, lipid arc >180°, and macrophage infiltration were associated with cardiac death or myocardial infarction (HR 7.54, 95% CI 3.1-18.6), cardiac death (HR 5.63, 95% CI 1.80-17.4), and myocardial infarction (HR 7.27, 95% CI 2.60-2.0.5).[102] In 1,474 patients enrolled in

the Massachusetts General Hospital OCT registry, lipid-rich plaques were associated with a higher rate of MACE compared with non-lipidic plaques at 4 follow-up (7.2% vs 2.6%; HR 1.98, 95% CI 1.05-3.73).[103] However, the difference in MACE was driven by VP revascularization, and percent area stenosis >68.5% showed the strongest association.[103] In another large registry, OCT-defined lipid-rich plaque (maximum lipid arc >180°) and TCFA (minimum thickness <65 μm) were associated with a higher incidence of ACS at a median follow-up of 6 years (HR 12.67, 95% CI 6.82-23.57 and HR 10.41, 95% CI 6.48-16.73, respectively).[104] The combination of the two high-risk features (4% of patients) improved the positive predictive value of OCT-defined VP.[104] Another study assessed the OCT-based morphologic predictors between slow linear and rapid step-wise plaque progression.[105] In this study, lipid-rich plaque, TCFA, and layered plaque were predictors of subsequent rapid plaque progression, and 61% of plaques with rapid progression at OCT follow-up showed a layered pattern as a plausible signature of previous plaque disruption and healing.[105] More recently, a study of 883 patients who underwent primary PCI showed that 4-year non-culprit lesion-related MACE were 2-fold higher compared with those culprit lesion-related and, TCFA and a MLA <3.50 mm^2 were independently associated with MACE after multivariable adjustment (HR 7.64, 95% CI 3.42-17.09, and HR 4.11, 95% CI 1.72-9.82, respectively).[106] The coexistence of the two morphological features improved the positive predictive value.[106]

An important limitation of previous intravascular imaging-based studies was the absence of functional assessment of non-culprit VPs. In this context, the frequent observation of low MLA values among the predictors of MACE may indicate that variable proportions of angiographically non-significant VPs were flow-limiting and some VP-related MACE were eventually unrelated to plaque destabilization. In the COMBINE OCT-FFR study, diabetic patients undergoing PCI with one or more fractional flow reserve-negative lesions were classified according to the presence or absence of OCT-defined TCFAs.[107] At 18 months, fractional flow reserve-negative TCFA-positive patients (n=98, 25%) experienced more frequently MACE (HR 4.65, 95% CI 1.99-10.89) compared with

those fraction flow reserve-negative TCFA-negative (n=292, 75%), and TCFA identification was the strongest predictor of MACE at follow-up (HR 5.12, 95% CI 2.12-12.34).[107]

Near-Infrared Spectroscopy

Near-infrared spectroscopy (NIRS), an established method in physical sciences to characterize the chemical composition of various biomaterials, was introduced as a catheter-based system to complement IVUS information and overcome its inherent technical limitations in detecting some VP characteristics.[24, 26] NIRS relies on the spectroscopic analysis of the light absorbed and backscattered off the tissues to provide a color-coded representation (chemogram) of the arterial wall cholesterol levels.[26] NIRS overtakes IVUS and OCT in detecting lipid-rich plaques and quantifying the lipid content by determining the maximal proportion of yellow pixels relative to the total number of pixels multiplied by 1000 into target segments, primarily of 4 mm (maximal lipid core burden index in 4 mm section, $maxLCBI_{4mm}$).[108]

The Lipid-Rich Plaque (LRP) study prospectively enrolled patients who underwent successful NIRS-IVUS-guided PCI of non-culprit segments scanning.[29] Among 1271 analyzable patients, the 2-year MACE cumulative incidence related to non-culprit lesions was 9% (n=103), to culprit lesions was 9% (n=104), and to indeterminate lesions was 2% (n=28).[29] In a plaque-level multivariable-adjusted model, $LCBI_{4mm}$ >400 (HR 3.39, 95% 1.85-6.20), plaque burden ≥70% within $maxLCBI_{4mm}$ (HR 3.99, 95% 1.38-11.56), and MLA ≤4 mm² within $maxLCBI_{4mm}$ (HR 1.79, 95% 1.02-3.16) were independently associated with a higher occurrence of 2-year non-culprit lesion-related MACE.[29] Later, the PROSPECT II study, including 898 patients with recent myocardial infarction and NIRS-IVUS assessment of non-culprit lesions, demonstrated in lesion level-analyses that maxLCBI4mm >324.7 (OR 7.47, 95 CI% 3.93-14.20), plaque burden ≥70% (OR 11.37, 95% CI 5.60-23.11), and MLA ≤4.0 mm2 (OR 4.99, 95% CI 2.61-9.54) were independent predictors of MACE.[30] Although two-thirds of events were progressive angina, the study was also unique in demonstrating predictive value for myocardial infarction alone. In the lesion-level multivariable model, only $maxLCBI_{4mm}$

>324.7 and plaque burden ≥70% remained statistically significant.[30] Unlike other studies on VPs, in PROSPECT II, patients with non-culprit lesion-related MACE (n=66, 8.0%) were about 2-fold higher than those related to the culprit lesion (n=36, 4.2%).[30] **Table 2** encompasses studies proving the association between the characteristics of untreated vulnerable coronary plaques assessed by mean of invasive techniques and long-term clinical outcomes.

Non-Invasive Techniques

Coronary Computed Tomography Angiography

Contemporary CCTA provides high spatial resolution information with diagnostic accuracy, limited procedural times, and low radiation and contrast media exposures.[36, 37] CCTA categorizes plaque composition and rupture risk primarily based on their density in Hounsfield Units (HU).[24, 35-37] In a study directly comparing CCTA with IVUS, a cut-off of 5.5% of pixels with an attenuation of <30 HU showed a 95% sensitivity and 80% specificity for VP identification.[109] Other studies confirmed this finding and showed that greater low attenuation plaque volume and percent of low attenuation plaque area referred to the total plaque area were independently associated with a higher incidence of ACS at long-term follow-up.[110, 111] The presence of plaques with low attenuation in the center (necrotic core) and an outer eccentric rim of high attenuation (fibrous cap) ("napkin-ring sign"), CCTA-defined positive remodeling (lesion diameter/reference diameter ≥10% larger than the proximal and distal reference segments), and spotty calcification (<3 mm) emerged among the strongest predictors of ACS.[110, 111] The SCOT-HEART study assessed the high-risk characteristics of positive remodeling, low attenuation plaque, spotty calcification, and napkin-ring sign in 1769 patients with CCTA images suitable for analysis.[112] Patients with plaques showing at least one risk feature had an approximately 3 times higher risk of cardiac death or nonfatal myocardial infarction compared with those without coronary artery disease or with plaques without risk features (HR 3.01, 95% CI 1.61-5.63).[112] Consistently, in the ROMICAT II trial, patients randomized to CCTA with plaques showing at least one high-risk feature among positive remodeling, low attenuation plaque,

spotty calcification, and napkin-ring sign, experienced more frequently an ACS (OR 8.9, 95% CI 1.8-43.3) compared with those without plaques showing high-risk features.[113] In the ICONIC study, most ACS precursor plaques were non-obstructive and, in the multivariable lesion-level analysis, percent diameter stenosis, percent cross-sectional plaque burden, fibrofatty and necrotic core volume, and the presence of at least 2 high-risk features among positive remodeling, low attenuation, and spotty calcification were associated with ACS at follow-up.[114] The study also showed that three-fourths of ACS precursor plaques showed <50% stenosis and only 31.0% showed at least 2 high-risk features.[114]

Overall, available CCTA studies generally define VP in the presence of at least one high-risk feature and there is limited data on the comparative impact of individual morphologic features, some of which do not present sufficient specificity to identify VP. For these reasons, the CAD-RADS 2.0 suggested defining VP in the presence of at least two high-risk features.[37] Moreover, given that available studies on CCTA often remain elusive in establishing a direct association between plaque rupture and cardiovascular events during follow-up, the predictive value of CCTA tends to be oriented toward the global assessment of coronary artery disease rather than individual VPs. In this context, the detection of the emerging imaging biomarker of perivascular fat attenuation and the additive value of artificial intelligence-enabled quantitative and hemodynamic analysis by CCTA has shown to enhance the identification of patients with coronary inflammation and higher risk of coronary artery disease destabilization.[115]

Magnetic Resonance Imaging

Currently, MRI presents some technical limitations for defining coronary artery disease compared with CCTA, Nevertheless, some studies have shown an high potential for identifying VPs and providing valuable prognostic information.[36] Indeed, MRI can detect intraluminal stenosis and plaque composition through different contrast weightings, including T1, T2, and proton density.[38] In some studies, MRI distinguished specific vulnerability indicators, including the fibrous cap, necrotic core,

macrophage infiltration, positive remodeling, hemorrhage, and neovascularization, and hyper-intense plaques on T1-weighted imaging indicated an high risk of rupture and thrombosis.[38]

Positron Emission Tomography

PET offers valuable insights for identifying and characterizing VPs and the promising findings may be bolstered when integrated with the diagnostic capabilities of CCTA or MRI.[39, 116] A variety of radionuclide molecules that have traditionally been used to detect metabolically active lesions can be used to identify characteristics correlated to plaque vulnerability including activated macrophage glucose consumption (^{18}F-fluorodeoxyglucose), apoptosis (technetium-99m-annexin V), microcalcifications in necrotic cores (^{18}F-sodium fluoride), neoangiogenesis (18F-galacto RGD), and targeted macrophage activity (^{68}Ga-DOTATATE).[24, 39, 116] However, further research is warranted to define the prognostic improvements of PET-based VP identification versus other established methods.

Table S1. Randomized Clinical Trials and Imaging Substudies of Randomized Clinical Trial of Lipid-Lowering Pharmacotherapy for the Atherosclerotic Plaque.

Trial (Year)	Sample size	Population	Imaging	Intervention	Control	Treatment duration (weeks)	Primary endpoint	Results (mean, 95%CI)
**REVERSAL, 2004**	654	Target vessel lesion > 20% and < 50% in a segment > 30 mm	IVUS	Atorvastatin 80 mg	Pravastatin 40 mg	72	Change in percent atheroma volume	-1.2 (-2.63 to 0.20) vs -4.2 (-5.2 to -2.9); p=0.01
**ASTEROID, 2006**	349	Target vessel lesion > 20% and < 50% in a segment > 40 mm	IVUS	Rosuvastatin 40 mg	-	96	Change in percent atheroma volume	-0.79 (*-1.21 to -0.53); p<0.001
							Change in atheroma volume in most diseased 10-mm subsegment	-5.6 (-6.82 to -3.96); p<0.001
**SATURN, 2011**	1039	Target vessel lesion > 20% and < 50%	IVUS	Atorvastatin 80 mg or	Rosuvastatin 40 mg	104	Change in percent atheroma volume	0.99% (−1.19 to −0.63) vs. 1.22% (−1.52 to −0.90); p=0.17
**YELLOW, 2013**	87	MVD undergoing PCI with ≥1 non-target lesion	NIRS and IVUS	Rosuvastatin 40 mg	Standard of care	7	Change in $maxLCBI_{4mm}$	-149.1(-210.9 to -42.9) vs 2.4 (-36.1 to 44.7); p=0.01
**EASY-FIT, 2014**	60	UA with untreated dyslipidemia and intermediate non-culprit lesion	OCT	Atorvastatin 20 mg	Atorvastatin 5 mg	52	Change in minimal FCT (µm)	73 (28 to 113) vs 19 (-1 to 48); p<0.001
**PRECISE-IVUS, 2015**	246	CAD and LDL-C>100 mg/dl	IVUS	Atorvastatin plus ezetimibe	Atorvastatin	52	Change in percent atheroma volume	-1.4 (-3.4 to -0.1) vs -0.3 (-1.9 to 0.9); p=0.001

**IBIS-4, 2015**	103	Non-IRA in patients with STEMI	IVUS	Rosuvastatin 40 mg	-	52	Change in percent atheroma volume	−0.9% (−1.56 to −0.25); p=0.007
**GLAGOV, 2016**	968	Target vessel lesion ≤ 50%	IVUS	Evolocumab plus statin	Placebo plus statin	76	Change in percent atheroma volume	-0.95 (-1.33 to -0.58) vs 0.05 (-0.32 to 0.42); p<0.001
**ODYSSEY J-IVUS, 2019**	206	ACS, >1 non-culprit lesion, and LDL-C>100 mg/dl	IVUS	Alirocumab plus statin	Standard of care	36	Change in total atheroma volume	-4.8 (1.0) vs -3.2 (1.0); p=0.228
**EVAPORATE, 2020**	80	Target vessel lesion > 20%, on statins and with elevated triglycerides	CCTA	icosapent ethyl plus statin	Placebo plus statin	72	Change in low-attenuation plaque	−0.3 ± 1.5 vs. 0.9 ± 1.7 mm3; p = 0.006
**HUYGENS, 2022**	161	NSTEMI, Untreated non-culprit vulnerable plaque	OCT	Evolocumab plus statin	Placebo plus statin	52	Change in minimal FCT (μm)	42.7 (32.4 to 53.1) vs 21.5 (10.9 to 32.1); p=0.015
							Change in maximum lipid arc (degrees)	-57.5 (-72.2 to -42.7) vs -31.4 (-50.2 to -12.7); p = 0.04
**PACMAN-AMI, 2022**	300	Patients undergoing PCI for acute MI and untreated, non-culprit lesions	NIRS, IVUS, and OCT	Alirocumab plus statin	Placebo plus statin	52	Change in percent atheroma volume	-2.13 (-2.53 to -1.73) vs -0.92 (-1.28 to -0.56); p<0.001

CAD=Coronary Artery Disease; CCTA=Coronary Computed Tomography Angiography; CI=Confidence Interval; FCT=Fibrous Cap Thickness; IVUS, Intravascular Ultrasound; $maxLCBI_{4mm}$=Maximum Lipid Core Burden Index in 4mm Lesion Segments, LDL=Low-Density Lipoprotein; MI=Myocardial Infarction; MVD=Multivessel Disease; NIRS=Near-Infrared Spectroscopy; NSTEMI=Non-ST Segment Elevation Myocardial Infarction; LDL-C=Low-Density Lipoprotein Cholesterol; OCT=Optical Coherence Tomography; PCI=Percutaneous Coronary Intervention; STEMI=ST-segment elevation myocardial infarction; UA=Unstable Angina.
* 97.5% confidence interval
